# Embryotoxicity and mixture effects of legacy PFAS in a human iPSC-based 3D model

**DOI:** 10.1007/s10565-026-10137-8

**Published:** 2026-01-19

**Authors:** Andreas Frederik Treschow, Elisa Martiny, Claudia Torero Gutierrez, Agnieszka Anna Niklas, Martin Scholze, Anne Marie Vinggaard, Maria João Valente

**Affiliations:** 1 National Food Institute, Technical University of Denmark, Kgs. Lyngby, 2800 Denmark; 2 Centre for Pollution Research and Policy, College of Health, Medicine and Life Sciences, Brunel University London, Kingston Lane, Uxbridge, UB8 3PH UK

**Keywords:** PFOS, PFOA, PFNA, PFHxS, Embryotoxicity, Mixture toxicity, Cardiomyocyte, hiPSCs, PFAS uptake

## Abstract

Humans are continuously exposed to a wide array of exogenous chemicals via dietary intake, environmental sources, and the use of personal care products. This includes per- and polyfluoroalkyl substances (PFAS), a class of highly persistent compounds that have been associated with developmental effects in humans. This study assessed the effects of four legacy PFAS, namely PFOS, PFOA, PFNA and PFHxS, and mixtures thereof in the PluriLum assay, a 3D human induced pluripotent stem cell (hiPSC)-based model for embryotoxicity testing. We established the individual embryotoxic potencies of PFAS, with PFNA exhibiting the highest potency, followed by PFOS, PFOA and PFHxS. The four PFAS were evaluated in three reconstituted mixtures, prepared either to reflect identical potencies (“equipotent mixture”) or the average serum concentrations reported for the European adult or child population (“real-life mixtures”). Comparing observed *versus* predicted mixture responses demonstrated concentration additivity throughout the entire range of tested concentrations. Studies on uptake in 3D embryoid bodies revealed the highest bioaccumulation of PFOS, followed by PFNA, PFOA, and PFHxS. Moreover, less than 2% of the nominally added PFAS could be recovered in the embryoid bodies. RNA sequencing showed that relatively few genes were affected by PFOS, PFNA and PFOA, however expression of genes related to focal adhesion and functional pathways associated with cardiac, cardiomyocyte and muscle tissue development was significantly changed. Notably, PFOS affected the greatest number of embryonic development pathways. In conclusion, the four tested PFAS significantly impaired cardiomyocyte differentiation, indicating embryotoxicity. The combined responses were consistent with the concentration addition principle, supported by shared functional pathways and indicative of common sites of molecular action.

**Supplementary information:**

The online version contains supplementary material available at 10.1007/s10565-026-10137-8.

## Introduction

Per- and polyfluoroalkyl substances (PFAS) are a class of thousands of synthetic chemicals characterized by wide variety, high persistence, mobility and stability, leading to bioaccumulation in the environment and living organisms. Due to their widespread use and presence in the environment, humans are exposed to a variety of PFAS through food, drinking water, and consumer products (Schrenk et al. 2020; Brunn et al. 2023). These compounds resist metabolic degradation and are slowly excreted, some displaying human half-lives of several years (Schrenk et al. 2020; Abraham et al. 2024). Human biomonitoring data consistently detect four PFAS, perfluorononanoic acid (PFNA), perfluorooctanoic acid (PFOA), perfluorooctanesulfonic acid (PFOS), and perfluorohexanesulfonic acid (PFHxS), in nearly all individuals (Schrenk et al. 2020). These four PFAS also show the highest serum concentrations in the adult European population (Schrenk et al. 2020; Abraham et al. 2024).

Although PFOS and PFOA have been banned in many countries, including the US and EU, PFAS continue to pose health risks due to their continued presence and the vast number of compounds in this class, more than 12,000 compounds (Brunn et al. 2023). Prenatal exposure to external substances can irreversibly disrupt normal foetal development (Zhao et al. 2024) and epidemiological studies have linked prenatal exposure to PFAS to a range of adverse health outcomes, including reduced birth weight, weakened immune function, impaired neurodevelopment, lowered bone mineral density, and endocrine disorders (Washino et al. 2009; Schrenk et al. 2020; Zhao et al. 2024; Luo et al. 2024). This underscores the urgent need to assess potential adverse effects of PFAS on human embryonic development, particularly in the context of early life exposure (Zhao et al. 2024). Given that humans are exposed to multiple PFAS simultaneously, evaluating their combined or mixture effects is essential. As the assessment of new PFAS and mixtures is demanding, there is a growing need for efficient, relevant, and high-throughput tools to evaluate both individual and mixture toxicity.

In this study, we investigated the embryotoxic effects of PFOS, PFOA, PFNA and PFHxS, as well as their mixtures. We applied our recently enhanced 3D reporter-gene assay, PluriLum, which is based on human induced pluripotent stem cells (hiPSCs). These cells form embryoid bodies (EBs) that are exposed to chemicals during their differentiation into beating cardio-spheres. In this hiPSC-based 3D EB model, we recapitulate early embryonic development beginning at the blastocyst stage (approximately 5–6 days post-fertilization in humans), progressing toward lineage specification and organogenesis. While cardiomyocyte differentiation serves as a measurable and biologically relevant endpoint, given that the heart is the first functional organ formed during embryogenesis, the model reflects broader embryotoxic potential, not solely cardiac bioactivity. In the PluriLum assay we measure activation of the cardiomyocyte-specific gene *NKX2.5* as a surrogate marker for cardiomyocyte differentiation. This gene peaks on day 6 of culture which corresponds to human gestational day 21–42 (Lauschke et al. 2021; Treschow et al. 2024a). We have previously demonstrated that this model is suitable for evaluating the embryotoxic potential of various chemical substances (Lauschke et al. 2021; Treschow et al. 2024a).

Furthermore, we used this test system to investigate the cellular uptake rates of the four PFAS and to explore the mechanisms of action through which they exert their effects.

## Materials and methods

### Reagents and chemicals

Materials used for the stem cell culture has been described previously (Lauschke et al. 2021; Treschow et al. 2024a). The cells were maintained in mTeSR™ Plus (STEMCELL Technologies Inc., Vancouver, Canada). Reagents including TrypLE™, Penicillin–Streptomycin-Glutamine (PSG), KnockOut™ DMEM medium, human fibroblast growth factor-basic (FGF2), and activin A, were purchased from Thermo Fisher Scientific Inc., (Massachusetts, USA). Sodium selenite, human transferrin, L-ascorbic acid 2-phosphate trisodium salt (Asc), PFNA (CAS: 375–95-1; > 97% purity), PFOA (CAS: 335–67-1; > 95% purity), PFOS (CAS: 2795–39-3; > 98% purity), ammonium acetate and 25% ammonia solution were purchased from Merck KGaA (Darmstadt, Germany). Abcam Plc (Cambridge, UK) was the supplier of rho kinase inhibitor. Corning® ITS Premix Universal Culture Supplement and Corning® Matrigel® hESC-Qualified Matrix were purchased from Corning Inc (New York, USA). Bio-Techne (Minnesota, USA) was the supplier of 4-(2-Methyl-4-pyridinyl)-N-[4-(3- yridinyl)phenyl]benzeneacetamide (Wnt-C59) and human bone morphogenetic protein 4 (BMP4). 6-(2-(4-(2,4-Dichlorophenyl)−5-(4-methyl-1H-imidazol- 2-yl)-pyrimidin-2-ylamino)ethyl-amino)-nicotinonitrile (CHIR99021) was purchased from Axon Medchem (Groningen, the Netherlands). PFHxS (CAS: 355–46-4; > 95% purity) was procured from Cayman Chemicals (BioNordika, Herlev, Denmark). Acetonitrile and methanol were procured from Honeywell (Seelze, Germany). Milli-Q water (resistivity 18.2 Ω·cm) was obtained using a Milli-Q Elix & QPOD purification system (Millipore). PFAS were quantified and identified using a set of 13 commercially available native standards together with 11 C^13^-isotopically labelled internal standards of analytical grade purity (> 98%) (Wellington Laboratories Inc. (Guelph, ON, Canada).

### Cell culture maintenance

The PluriLum assay was performed using the BIONi010-C-*NKX2.5*-T2A-*Nluc*−44.37 cell line, developed in partnership with Bioneer A/S (Horsholm, Denmark) (Lauschke et al. 2021). The cells were maintained in mTeSR™ Plus medium on Matrigel®-coated cell culture dishes (Thermo Fisher Scientific Inc., Massachusetts, USA) at 37 °C and 5% CO_2_ in a humid environment. The cells were subcultured approximately once a week by 0.02% EDTA dissociation. Passages 37–43 were utilized during the work.

### Cytotoxicity assessment

To assure the assessment of non-cytotoxic concentrations in the PluriLum assay, cell death induced by the single PFAS was first determined, as previously described in Lauschke et al. (Lauschke et al. 2020), with minor modifications. For this purpose, near confluent 2D hiPSC cultures were dissociated into single cells by using TrypLE™, seeded into Matrigel®-coated white 96-well plates at 1 × 10^4^ cells/well in mTeSR™ Plus medium + 1:100 PSG and 10 μM Y27632 dihydrochloride. Cells were allowed to attach, and after 24 h, the medium was replaced by fresh mTeSR™ Plus medium + 1:100 PSG. After 24 h further incubation the medium was removed, and cells were then exposed for 24 h to each of the single PFAS at a final concentration range of 0.01–200 µM in mTeSR™ Plus medium + 1:100 PSG. After 24 h of further incubation the medium was removed and new medium with the exposure chemicals were added for another 24 h. By the end of the exposure period (total 48 h), cell viability was assessed adding equivalent volumes of the CellTiter-Glo® Luminescent Cell Viability Assay (Promega, Wisconsin, USA), pipetting up and down and letting the plate rest for 20 min. A EnSpire 2300 Microplate Reader (PerkinElmer, Inc., Massachusetts, USA) was used to measure luminescence.

### Cardiomyocyte differentiation

The differentiation protocol was described in previous work (Treschow et al. 2024b). Briefly, after dissociation with TrypLE™, cells were seeded into 96-well Polystyrene Conical Bottom MicroWell™ plates (Thermo Fisher Scientific Inc., Massachusetts, USA) at a density of 5 × 10^3^ cells/well, and centrifuged at 500 *g* for 5 min on Day −1. The plates were incubated overnight for 19 h at 37 °C and 5% CO_2_, at which point EBs have formed. On Day 0 of the protocol, the culture medium was replaced with a differentiation medium based on Knock-out DMEM containing PSG, activin A, FGF2, BMP4, ITS, CHIR and rock inhibitor. After incubating for 24 h (Day 1), the medium was replaced by “TS medium” containing PSG, Asc and human transferrin and sodium selenite. After incubation for 24 h (Day 2), medium was replaced by WNT-containing TS medium. After 24 h (Day 3), the medium was replaced by TS medium and plates were incubated for 72 h until Day 6 of the protocol where the assay was terminated. This regimen allows differentiation of EBs to closely follow key developmental stages of the developing embryo: with loss of their pluripotency from Day 0, followed by formation of mesoderm, cardiac mesoderm, cardiac progenitors, and finally contracting cardiomyocytes by Day 6 (Lauschke et al. 2020). Termination of the protocol on Day 6 relies on the fact that the expression of the cardiac lineage marker *NKX2.5*, on which this reporter gene assay was built on, peaks at Day 6, providing improved sensitivity to the assay compared to readouts thereafter (Treschow et al. 2024b).

### Chemical exposures

All stock dilutions of individual PFAS and mixtures were prepared in DMSO at 1000-fold the final desired concentration on the plate. EBs were exposed on Days 1, 2 and 3, targeting the moment of shift from mesoderm to early cardiac differentiation, mimicking the earliest stages of embryo development (Lauschke et al. 2020). On each experimental day, chemical exposures were performed by diluting the DMSO stocks 1:1000 into the respective medium. A concentration of 0.1% DMSO (V/V) was maintained in all wells. During the hiPSC differentiation, EBs were exposed to either single PFAS or mixtures by adding them to the respective differentiation media on Days 1, 2 and 3. There was no medium change until termination on Day 6, making a total exposure time of 120 h. The DMSO concentration was kept at 0.1% during all the exposure time.

To capture effects across the full response range, eight concentrations of each PFAS were tested in the range of 0.1–200 µM for PFOA and PFHxS, 0.1–60 µM for PFOS, and 0.1–40 µM for PFNA. A 10% inhibition in luminescence at assay termination at Day 6 was defined as the benchmark response (BMR), representing the smallest consistent effect distinguishable from controls that enabled robust estimation of the benchmark concentration (BMC).

An equipotent mixture composed of four PFAS was prepared using their individual BMC_10_ values: 10.2 µM PFNA, 13.2 µM PFOS, 23.7 µM PFOA and 31.4 µM PFHxS, in total 78.5 µM. This mixture was tested across an enrichment range of 0.01–1.5, with an enrichment factor of 1 corresponding to the sum of the individual BMC_10_. Two additional “real-life” mixtures were designed based on average serum concentrations reported for PFNA, PFOS, PFOA, and PFHxS in European adults and children (Schrenk et al. 2020), and tested over an enrichment range of 0.1–2000. For the adult exposure scenario, the mixture at enrichment factor 1 consisted of 15.0 nM PFOS, 12.3 nM PFHxS, 5.1 nM PFOA and 1.6 nM PFNA (total: 34 nM), and for the children exposure scenario, enrichment factor 1 consisted of 6.6 nM PFOS, 1.4 nM PFHxS, 8.0 nM PFOA and 2.0 nM PFNA (total: 18 nM).

Overall, four individual PFAS and three different mixture compositions were tested at varying concentrations. For each treatment, six EBs were used.

### Imaging and size measurement of embryoid bodies

On Day 0 of the protocol, individual EBs were measured using a BioTek Cytation 5 Cell Imaging Multimode Reader (Agilent Technologies, Santa Clara, USA) with a 4× objective.

The BioTek Gen5 Image Prime Software was used for quality control of the average size of the EBs in the individual experiments. The control was performed by masking the edge of the spheroids and measuring the diameter of the individual EBs. Experiments with average EB sizes below 500 µm were discarded in accordance with our quality control step based on size threshold established in previous work (Treschow et al. 2024b).

### Analysis of *NKX2.5* activation by luminescence measurements

*NKX2.5* activation-derived luminescence in the individual spheroids was measured by the end of the differentiation protocol using the Nano-Glo® Luciferase Assay System (Promega, Wisconsin, USA). The procedure was performed as described in Treschow et al. (Treschow et al. 2024b). In brief, the spheroids were washed and 40 μL were transferred to flat bottomed white 96-well plates on Day 6. Forty μL of papain solution (40 U/ml) was then added to each well and incubated for at least 90 min at 37 °C. Following the incubation period, the spheroids were mechanically dissociated into a single-cell suspension through repeated pipetting. After dissociation, 40 μL of the cell suspension was added to a flat-bottomed white 96-well plate preloaded with 40 μL of Nano-Glo® Luciferase Assay Substrate. The contents were homogenized by repeated pipetting, and luminescence was subsequently quantified using an EnSpire 2300 Multimode Microplate Reader (PerkinElmer Inc., Massachusetts, USA).

### Concentration-effect analysis

All concentration-effect data were analyzed by normalizing luminescence readouts to the mean of the corresponding controls within each experiment. For statistical analysis, the mean of replicate-normalized values per experiment was used as the unit of analysis. Each individual PFAS and mixture was tested in three independent experiments, yielding pooled data sets of three effect values per control and exposure treatment. A best-fit approach was used to model concentration-effect relationships (Scholze et al. 2001). For each pooled data set, multiple nonlinear regression models were fitted, and the model with the best fit was chosen.

The principles of concentration addition (CA) and independent action (IA) were used for predicting the effects of the reconstituted mixtures, both assuming non-interaction between the mixture compounds (Greco et al. 1995). For CA, the concentration of the mixture required to cause an *x*% inhibition (*ICx*_*mixture*_) was calculated based on the individual *ICx* values (*ICx*_*i*_) of each individual compound *i* that produces the same response X, and their relative molar contribution *p*_*i*_ of each compound *i* to the mixture (Faust et al. 2003):

1 $${ICx}_{mixture}= {\left[\sum_{i=1}^{n}\left(\frac{{p}_{i}}{{ICx}_{i}}\right)\right]}^{-1}$$

IA can be defined for a mixture of *n* components by:

2 $${E(c}_{mixture})= 1- \prod_{i=1}^{n}\left(1-E\left({c}_{i}\right)\right)$$

where *E(c*_*i*_*)* denotes the effect caused by the individual compound *c*_*1*_ of the i^th^ compound, and *E(c*_*mixture*_*)* is the total effect of the mixture concentration *c*_*mixture*_.

A combined Monte-Carlo (MC) and nonlinear regression bootstrap simulation was used to determine approximate 95% confidence limits around the predicted mean response in order to account for statistical uncertainty in the mixture predictions. Parametric bootstrapping was used to simulate a distribution of resampled model fits for each compound, with resamples taken from the fitted nonlinear effect model (Faust et al. 2003). The MC simulation was then run using these as input to generate a distribution of predicted mixtures responses. When the 95% confidence intervals of the predicted and experimentally observed mixtures effects did not overlap, differences between the two were considered statistically significant.

### PFAS cellular uptake

Following exposure to each single PFAS at 5 µM, spheroids were dissociated by papain treatment as described above. Cell suspensions from 18 spheroids from each condition were then pooled, centrifuged at 500 *g* for 5 min, washed with Dulbecco’s phosphate buffered saline, centrifuged again at 500 *g* for 5 min, and the supernatant was finally discarded. Pelleted cells were then resuspended in 500 µL of acetonitrile (ACN), ultrasonicated for 20 min, vortexed and solubilized by pipetting. Samples were then centrifuged at 500 *g* for 5 min, 400 µL of supernatant were collected for PFAS quantification by LC–MS/MS and stored at −20 ᵒC until further analysis. The remaining pellet in 100 µL of ACN was added to 900 µL of sterilized dH_2_O and was completely solubilized by pipetting up and down. These samples were stored at −20 ᵒC for total protein content analysis.

On Days 2 and 3, immediately before the next exposure timepoint, 80 µL of spent exposure media was collected from each well and stored at −20 ᵒC for PFAS quantification as well.

PFAS uptake was quantified using an LC–MS/MS method for PFAS in food (No. FC430) accredited according to ISO 17025. The LC–MS/MS instrumentation consisted of an Ultimate 3000 LC (Thermo Fisher Scientific, Waltham, MA, USA) coupled to EVOQ Elite triple (QqQ) MS/MS (Bruker Corporation, MA, USA). LC-separation was achieved using an Acquity UPLC CSH C18 column (130 Å, 1.7 μm, 100 × 2.1 mm; Waters Corporation, MA, USA). To separate any potential contamination originating the from LC system, a delay column (Acquity UPLC® BEH C18, 130 Å, 2.1 × 50 mm, 1.7 μm particle size) was used.

The eluents were: A) 2 mM ammonium acetate in Milli-Q water pH adjusted to 8.0, and B) methanol. The injection volume was 5 µL, with a column oven temperature of 50 ᵒC, and an autosampler temperature of 10 ᵒC. Detection of each PFAS was performed in MRM mode. Method description is presented in detail in Lerch et al. (Lerch et al. 2022).

The Bradford’s method (Bradford 1976) was used to determine total protein concentration of the samples. For this purpose, standard solutions of bovine serum albumin (2–200 g/mL) were prepared in 1:10 ACN:dH_2_O. In a 96-well clear bottom plate, 200 µL of Bradford reagent were added to 40 µL of diluted samples and standards per well, in triplicates. The plate was shaken at 350 rpm for 10 s at room temperature, and absorbance was measured at 595 nm (reference reading at 690 nm) in a BioTek Cytation 5 Cell Imaging Multimode Reader (Agilent Technologies, Santa Clara, USA).

LC–MS/MS quantification results, in ng/mL, were normalized to total protein content, in µg/mL. Data on PFAS levels from three independent experiments are presented in ng PFAS/µg protein. Statistical analysis was performed using GraphPad Prism 10 (version 10.4.2) for Windows. Multiple comparisons were performed through one-way ANOVA analysis, followed by Bonferroni’s post hoc test. Significance was accepted for *p* < 0.05. Relative uptake of PFAS was calculated using PFOA levels as the reference chemical, set as 1.

### RNA extraction

For RNA-sequencing, EBs were exposed to single PFAS at their BMC_10_ levels, and samples were collected on Day 6 of differentiation. RNA was extracted from a pool of 24 EBs per condition using the RNeasy Micro Kit (cat. No. 74004) (Qiagen, Hilden, Germany). A slight modification to the manufacturer’s instructions was made by reducing the amount of RNeasy Lysis Buffer used (75 µL instead of 350 µL), to avoid contamination of RNA with guanidinium thiocyanate. The remainder of the extraction was performed according to the manufacturer’s specifications. RNA with an A260/A280 ratio > 1.8 were measured for all samples extracted.

### RNA sequencing and analysis

Novogene Europe (Cambridge, UK) performed RNA sequencing, library preparation, and bioinformatics analysis on three biological replicates. mRNA was purified from total RNA by poly-T oligo-attached magnetic beads for directional and non-directional library cDNA synthesis. Library quality was assessed with a Qubit™ 2.0 fluorometer (Thermo Fisher Scientific, Massachusetts, USA), quantified via RT-PCR, and evaluated for size distribution using a bioanalyzer. Sequencing was conducted on the Illumina platform. Alignment and statistical methods to detect differentially expressed genes (DEGs) were performed by Novogene.

Raw reads were processed with Fastp to remove adapter, poly-N, and low-quality reads, while sequencing quality metrics (Q20, Q30 and GC content) were calculated. Reads were aligned to reference with HISAT v2.0.5 (Mortazavi et al. 2008) and gene expression quantification used featureCounts v1.5.0-p3 (Liao et al. 2014). Fragments Per Kilobase of transcript sequence per millions of base pairs sequenced (FPKM) (Trapnell et al. 2010) was calculated based on read counts and length of a gene to estimate gene expression levels. Principal Component Analysis was performed on the FPKM, and a batch correction was performed using the Combat function from the sva R package (Leek et al. 2012). Differentially expressed gene analysis was performed using the DESeq2 package (Love et al. 2014) to adjust read counts through one scaling normalized factor, and *p*-values were adjusted using the Benjamini and Hochberg method. Gene ontology (GO) enrichment analysis was performed using the clusterprofiler R package (Yu et al. 2012) with gene length bias corrected. GO terms with adjusted *p*-values below 0.05 were considered significantly enriched.

A Venn diagram showing the overlap of DEGs (adjusted *p-value* < 0.05; |log2FoldChange|> 1) between the four PFAS treatments was prepared using the VennDiagram R package (Chen and Boutros 2011). Significantly enriched GO terms deemed relevant under the scope of the assay were filtered based on their association to embryogenesis and cardiogenesis, and clustered using Cytoscape, ClueGo and CluePedia (Bindea et al. 2009). The feature *Fusion* was used to reduce redundancy concerning parent–child terms. Overlap in GO terms was visualized using a dot plot in R. Raw and processed data, along with the R script, is available in: https://zenodo.org/records/17600724.

### RT-qPCR analysis

To validate the quality of the RNA-sequencing data, five genes were selected for mRNA expression analysis by real time-quantitative PCR (RT-qPCR). Genes were selected based on Log2FoldChange and on their relation to embryonic and cardiac development (Table 1). For this purpose, the isolated RNA was diluted and converted to cDNA using the Omniscript RT kit from Qiagen (Hilden, Germany) with the addition of random hexamers mix (New England Labs, Massachusetts, USA). cDNA samples were further diluted into a 96-well plate. TaqMan™ gene expression assays (Thermo Fisher Scientific Inc., Massachusetts, USA) were used for RT-qPCR analysis of a total of seven genes, including the two housekeeping genes, *ACTB* and *GAPDH*, which were used to normalize the data. The gene expression assays are commercially preformulated with two unlabeled PCR primers and one FAM dye labeled TaqMan MGB probe. A total of 7.5 ng of cDNA per sample was analyzed in duplicates. Gene expression was quantified relative to negative control and housekeeping gene using the 2^−ΔΔCT^ method (Livak and Schmittgen 2001). Data were obtained from three independent experiments, run in duplicates. Statistical analysis was performed using GraphPad Prism 10 (version 10.4.2) for Windows. Comparisons between mRNA expression levels of each gene in EBs treated with PFAS and negative control were performed by unpaired t-Test. Significance was accepted for *p*-values < 0.05.

**Table 1:** Selected genes for RT-qPCR confirmation of RNA sequencing analysis

Gene	Assay ID	Encoded protein	Protein function
*ALDH1L2*	Hs01105342_m1	Mitochondrial 10-formyltetrahydrofolate dehydrogenase	Catalyzes the conversion of 10-formyltetrahydrofolate to tetrahydrofolate and CO_2_
*MYH2*	Hs00430042_m1	Myosin-2	Motor molecule with ATPase activity essential for muscle contraction
*TBX18*	Hs01385457_m1	T-box transcription factor TBX18	Transcriptional repressor involved in developmental processes of several tissues/organs, including the heart; essential for embryonic development of the sino atrial node head area, responsible for initiating the heartbeat
*TTN*	Hs00399225_m1	Titin	Component of the assembly and functioning of striated muscles in vertebrates
*RSPO1*	Hs00543475_m1	R-spondin-1	Activator of the canonical Wnt signaling pathway
*ACTB* (housekeeping)	Hs01060665_g1	β-actin	Cytoskeletal actin involved in cell motility, structure, contraction and intercellular signaling
*GAPDH* (housekeeping)	Hs02786624_g1	Glyceraldehyde-3-phosphate dehydrogenase	Enzyme that catalyzes the first step of the glycolysis pathway

## Results

### Cardiomyocyte differentiation

**Fig. 1:**
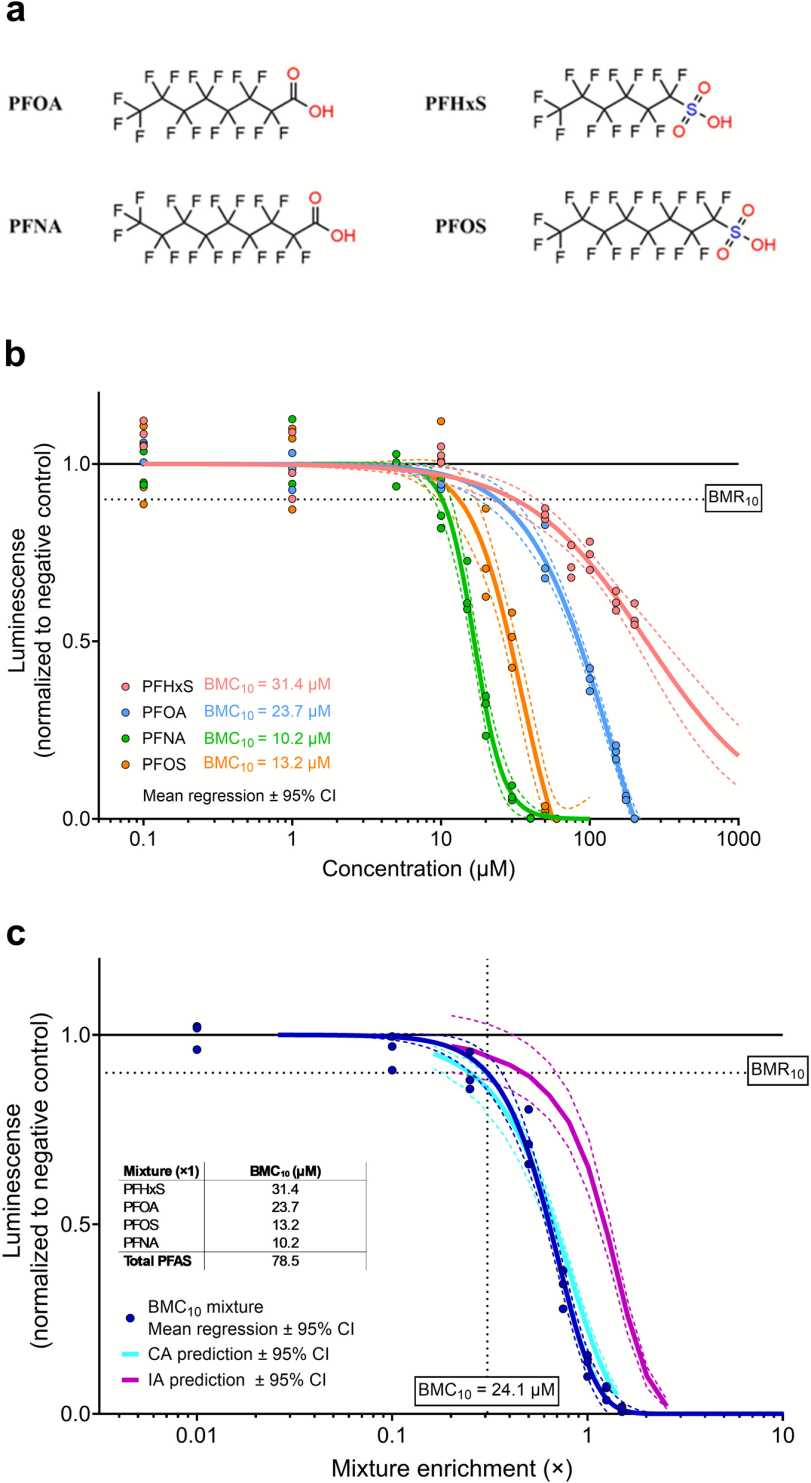
Effects of four PFAS in the PluriLum assay and predicted responses. **a** Chemical structures of the four PFAS under study: PFOA, PFNA, PFHxS, and PFOS. **b** Concentration-effect data of PFOA, PFNA, PFOS and PFHxS in the PluriLum assay, showing reduced *NKX2.5* activation measured as reduced luminescence. Experimental effect data are shown as average effect responses per experiment where each dot represents means from six EBs. Best-fitting nonlinear regression fits are shown as mean ± 95% confidence intervals (solid and dashed lines). Horizontal dotted line represents the benchmark response (BMR) of 10%, with corresponding benchmark concentrations (BMC_10_) for each single PFAS shown in the left lower corner. **c** Experimental and predicted responses of a mixture composed of four PFAS at their BMC_10_ values. The enrichment factors on the x-axis are calculated relative to the individual BMC_10_ values (set as enrichment factor of 1), which are shown in the embedded table. Experimental effect data are shown as average effect responses per experiment where each dot represents six EBs. The best-fitting nonlinear regression fits are shown as mean ± 95% confidence intervals (solid and dashed lines). Horizontal dotted line represents the benchmark response (BMR) of 10%, with corresponding benchmark concentrations (BMC_10_) of 24.1 µM for the PFAS mixture shown at the bottom of the vertical dotted line

Exposure to PFNA, PFOS, PFOA and PFHxS (Fig. 1a) led to reduced cardiomyocyte differentiation in the PluriLum assay, as indicated by a reduced total *NKX2.5* transcription on Day 6 (Fig. 1b). Among the compounds, PFNA showed the highest potency, followed by PFOS, PFOA and PFHxS. The benchmark concentration causing a 10% reduction in differentiation (BMC_10_) was 10.2 µM for PFNA, 13.2 µM for PFOS, 23.7 µM for PFOA, and 31.4 µM for PFHxS. None of the tested PFAS induced cytotoxicity in 2D cultures of undifferentiated hiPSCs following 48-h exposure at concentrations up to 200 µM (data not shown).

Using the BMC_10_ of PFOA as a reference, the relative potencies in the PluriLum assay were 1: 2.3: 1.8: 0.8 for PFOA, PFNA, PFOS, and PFHxS, respectively. When based on the IC₅₀ values (83, 16, 29, and 230 µM, respectively), the relative potencies were 1: 5.2: 2.9: 0.4, respectively.

### Concentration effect analysis

A mixture of PFNA, PFOS, PFOA, and PFHxS was prepared at their respective BMC_10_ concentrations for reduced *NKX2.5* activation and tested in the PluriLum assay using fixed-ratio dilutions (Fig. 1c). Mixture modelling based on concentration–effect curves for the individual PFAS was conducted using both CA and IA. The observed mixture effects aligned closely with CA predictions across the entire effect range, while IA consistently underestimated the mixture response. (Fig. 1c).

**Fig. 2:**
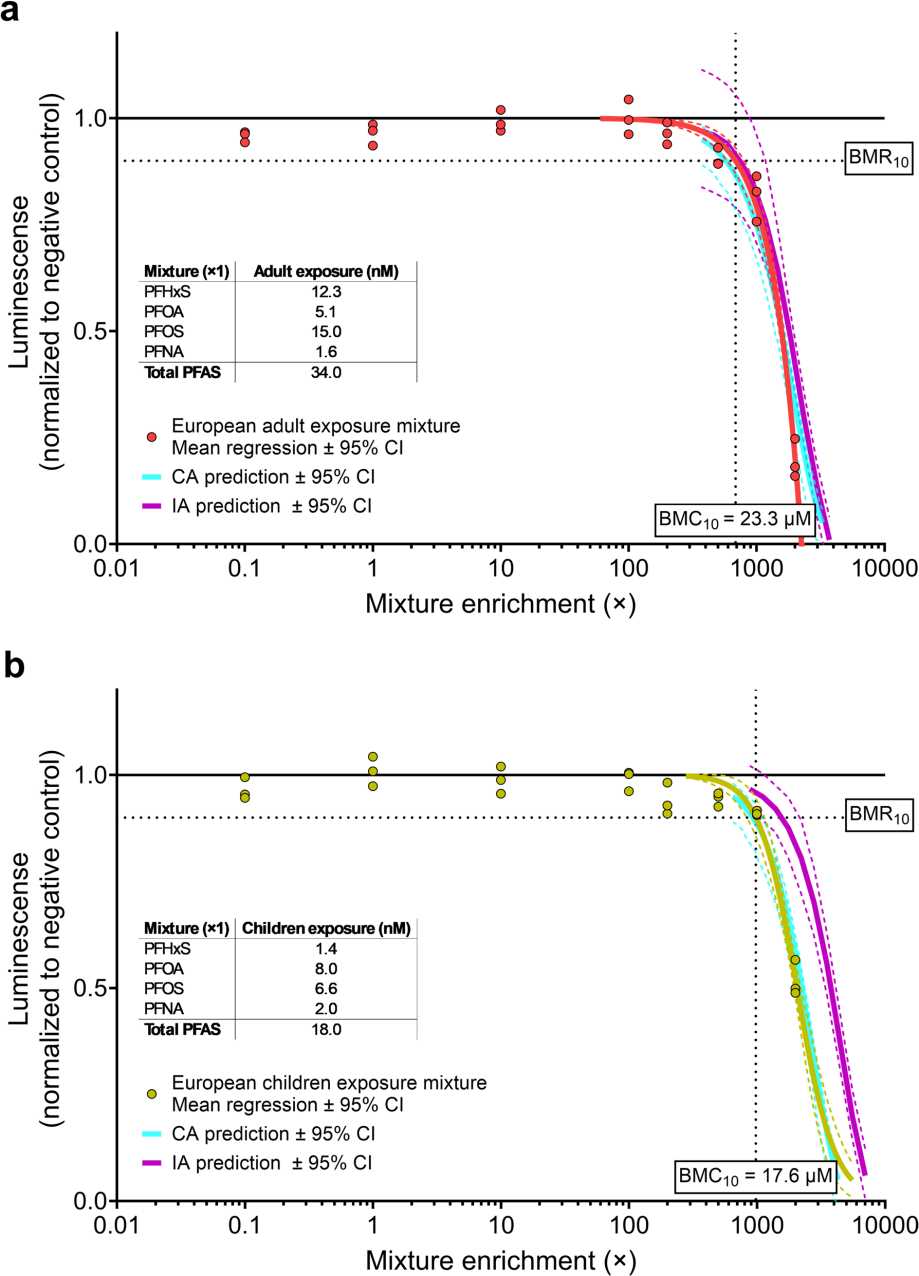
Observed and predicted responses of a mixture composed of four PFAS according to reported average serum levels of **a** European adults, and **b** children by EFSA (Schrenk et al. 2020). The enrichment factors on the x-axis are calculated as the nominal concentrations relative to the actual average exposure as reported by EFSA (set as an enrichment factor of 1), which are shown in the embedded tables. Experimental effect data are shown as average effect responses per experiment where each dot represents six EBs. The best-fitting nonlinear regression fits are shown as mean ± 95% confidence intervals (solid and dashed lines). Horizontal dotted lines represent the benchmark response (BMR) of 10% for the mixtures experimental data, with corresponding benchmark concentrations (BMC_10_) shown at the bottom of the vertical dotted line

Additionally, two mixtures reflecting average serum concentrations reported for European adults and children were tested at increasing enrichments levels. As expected for environmentally relevant exposure mixtures, the contribution of individual PFAS was highly unbalanced, with PFOS being the dominant compound in adults’ serum and PFOA in children’s serum. Consequently, predicted CA and IA curves were very similar, making it challenging to differentiate between the models. Nonetheless, CA provided the best fit to the observed mixture effects (Fig. 2). A 10% response (BMR₁₀) was observed at ~ 685-fold enrichment in the adult-based mixture (Fig. 2a), and at ~ 980-fold enrichment in the children-based mixture when comparing nominal PFAS concentrations to human exposure levels (Fig. 2b). Notably, these levels are highly exaggerated due to the low levels of free PFAS present in the EBs.

### PFAS cellular uptake

**Fig. 3:**
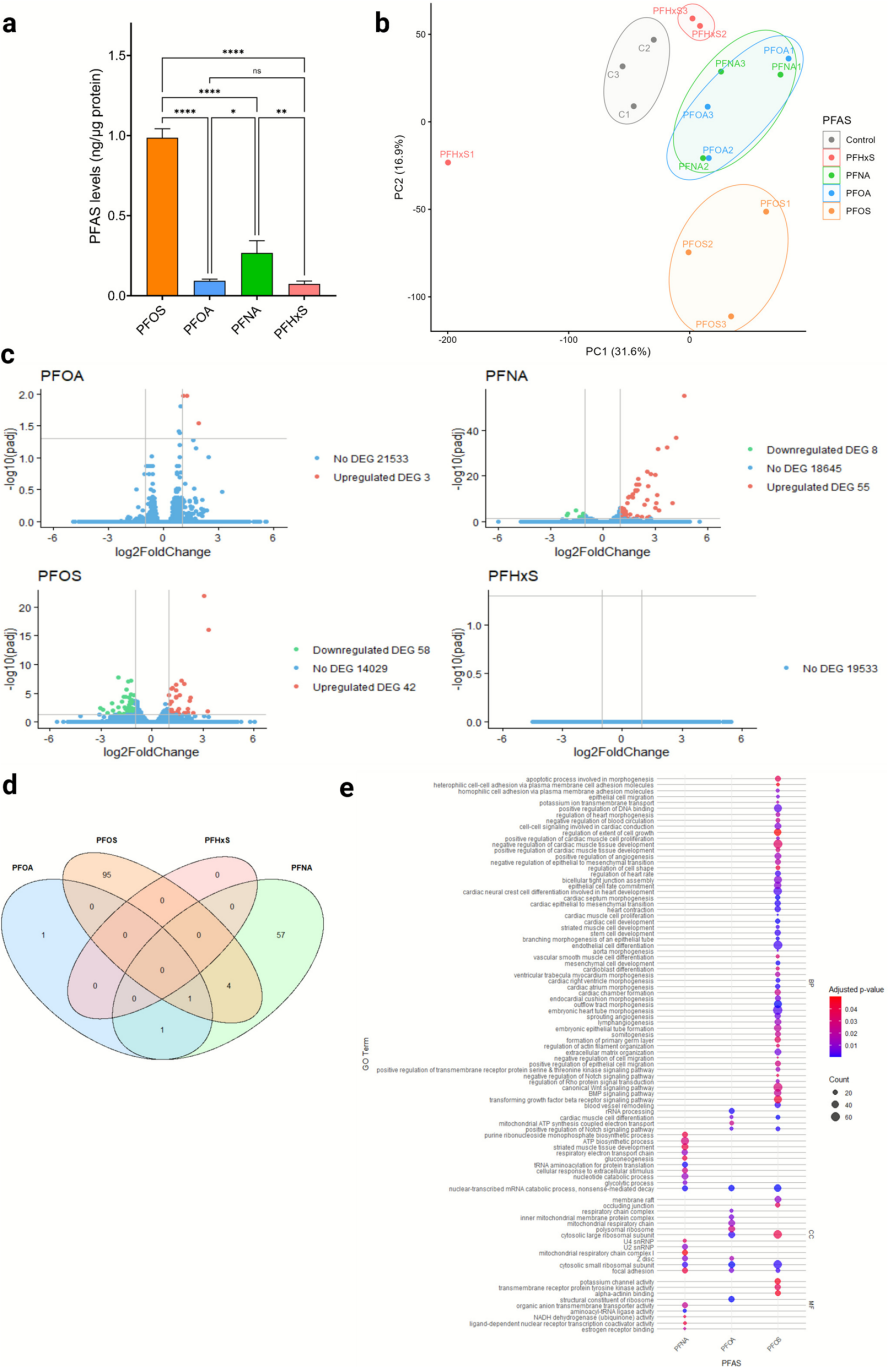
Cellular uptake and transcriptomic effects of PFAS. **a** Cellular uptake of PFOS, PFOA, PFNA, and PFHxS in the embryoid bodies of the PluriLum assay following exposure to 5 µM of each single PFAS. Experimental data are shown as average levels of PFAS normalized to total protein content. *p < 0.05, **p < 0.01, ****p < 0.0001. **b** Principal component (PC) analysis of transcriptomic profiles following treatment with each of the four PFAS at BMC_10_ on EBs at Day 6 of differentiation in the PluriLum assay. Analysis was performed on three biological replicates. **c** Volcano plots presenting upregulated (in red) and downregulated (in green) DEGs, along with genes that were not differentially expressed (in blue) after exposure to the four PFAS. DEG presented adjusted *p*-value < 0.05 and |Log_2_FoldChange|> 1. **d** Venn diagram depicting the overlap of DEGs. **e** Representation of a curated list of GO terms affected by exposure to PFOA, PFOS and PFNA in the PluriLum assay. The significance of adjusted *p*-values of each GO term is indicated by the colour scheme, while the dot size refers to the number of DEGs involved in each GO term. BP – biological processes, CC – cellular components, MF – molecular functions

When the EBs were exposed to individual PFAS at 5 µM (Fig. 3a), PFOS showed the highest cellular uptake (986 ± 56 pg/mg protein), significantly exceeding that of PFNA (269 ± 75 pg/mg protein, *p* < 0.001 *vs.* PFOS). PFOA (94 ± 10 pg/mg protein) and PFHxS (74 ± 19 pg/mg protein) exhibited lower uptake levels, which were not significantly different from each other. Relative to PFOA, the uptake ratios were 1: 2.9: 10.5: 0.8 for PFOA, PFNA, PFOS, PFHxS, respectively. Levels of the PFAS were also determined in Day 2 and Day 3 spent media and used to estimate the fraction of PFAS retained in the EBs by Day 6. We observed that the quantified levels of PFAS in the EBs represent a small percentage of the nominal exposure concentrations, with PFOS showing the highest cellular fraction of 1.7%, followed by PFNA with 0.7%, PFOA with 0.3% and finally PFHxS with merely 0.2% (Online Resource 1).

### RNA sequencing

To investigate the mechanisms of action underlying the effects of each PFAS at the BMC_10_ level on cardiomyocytes differentiation, RNA-sequencing was conducted on spheroids collected on Day 6 of the differentiation protocol. Principal Component Analysis (PCA) showed that the EBs exposed to the PFAS presented a different transcriptomic profile than the control group. Additionally, the PFOA and PFNA groups presented a similar transcriptomic profile, while one of the biological replicates from the PFHxS group presented a different profile than the other two biological replicates. The first principal component explained (PC1) 31.6% of the variance, while 16.9% of the variance was explained by the second principal component (PC2) (Fig. 3b). In total, PFOA presented three differentially expressed genes (DEGs), PFNA presented 63 DEGs, and PFOS presented 100 DEGs (Fig. 3c). PFHxS exposure did not result in any detectable gene deregulation. Some overlap was observed, with four DEGs shared between PFOS and PFNA, one between PFOA and PFNA, and one gene, *TTN,* encoding titin protein in striated muscle, deregulated by all three active PFAS (Fig. 3d). A full list of DEGs is provided in Online Resource 2.

To explore affected signaling pathways, GO enrichment analysis was conducted. This revealed 403 significantly enriched GO terms for PFOS, 142 for PFNA, and 62 for PFOA. No significant GO term was identified for PFHxS. The full GO term lists were curated to focus on those relevant to the assay, particularly those related to embryogenesis and cardiogenesis. The clustered results are shown in Fig. 3e and detailed in Online Resource 3.

The results demonstrate that PFOS significantly affected several biological processes (BP) directly related to embryonic and heart development, including cardiac cell development, stem cell development, and embryonic heart tube morphogenesis. It also affected extracellular matrix organization and the canonical Wnt signaling pathway. PFOA primarily impacted cellular components (CC), such as sarcomeres, the respiratory chain, and ribosomes, while PFNA affected pathways involving ATP biosynthesis and striated muscle development. Notably, all three PFAS affected focal adhesion, suggesting a potential common mechanism of action. Focal adhesions are dynamic structures that form at the interface between the cell and the extracellular matrix. They function as anchor points, physically linking the cell to the extracellular matrix via transmembrane proteins, and serve as critical hubs for intracellular signalling (Sarkar 1999).

**Fig. 4:**
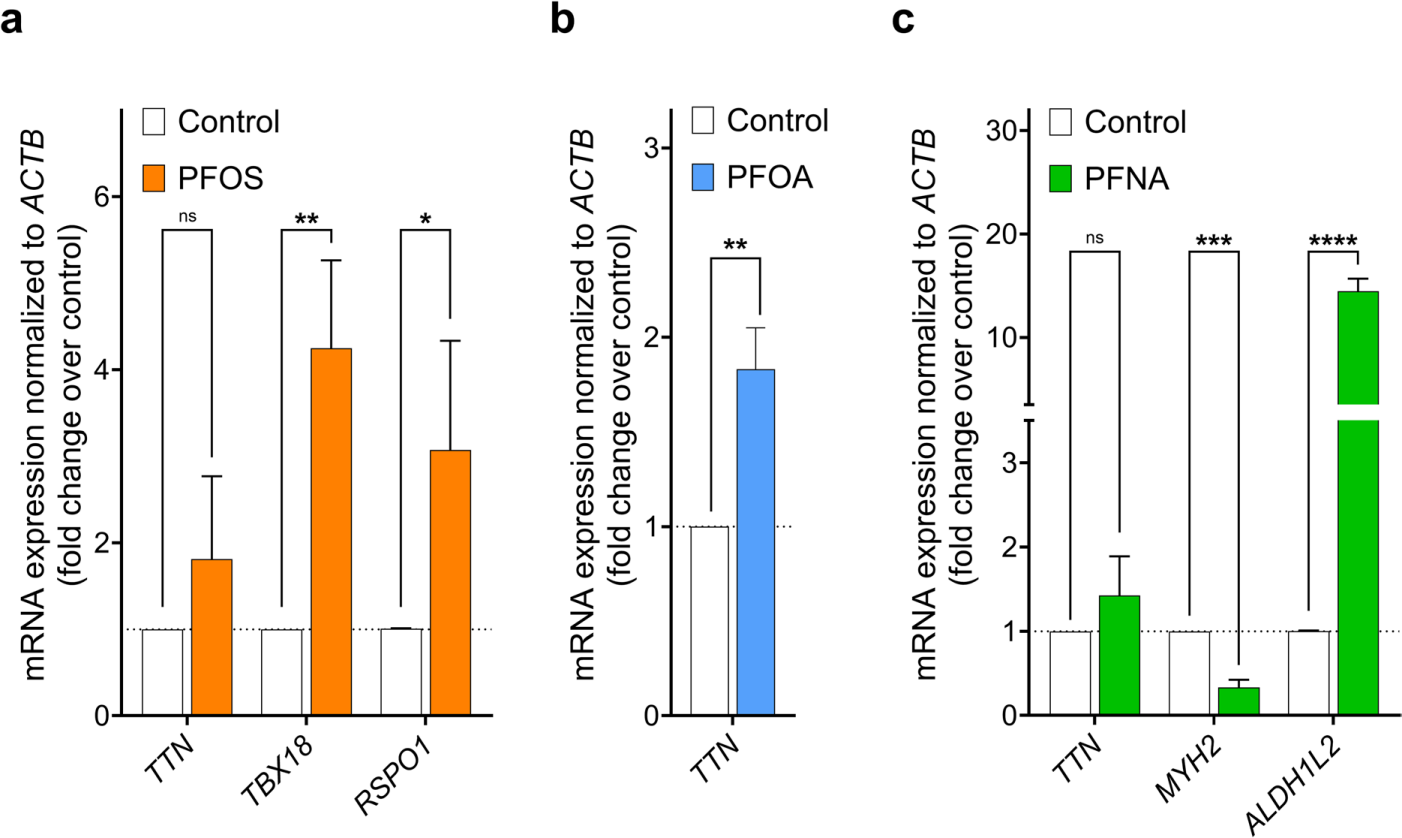
mRNA expression of genes selected for validation of the RNA-sequencing analysis. Experimental data is presented as average expression of each gene normalized to mRNA expression of the housekeeping gene *ACTB* (*n* = 3). *p < 0.05, **p < 0.01, ***p < 0.001, ****p < 0.0001

RT-qPCR analysis of selected DEGs, identified based on adjusted *p*-value < 0.05 and |Log_2_FoldChange|> 1, confirmed several of the RNA sequencing findings. Upregulation of *TTN*, observed in PFOS-, PFOA- and PFNA-treated EBs, was validated at the mRNA level (Table 2), reaching statistical significance only for PFOA (Fig. 4b, *p* < 0.01 vs. control). Similarly, *TBX18* and *RSPO1* were significantly upregulated in PFOS-treated cells (Fig. 4a; *p* < 0.01 and p < 0.05 vs. control, respectively). In PFNA-treated cells, *ALDH1L2* was markedly upregulated (Fig. 4c; *p* < 0.0001 vs. control), while *MYH2* expression was significantly downregulated (Fig. 4c; *p* < 0.001 vs. control).

**Table 2:** RNA sequencing analysis of selected genes for RT-qPCR confirmation

PFAS	Gene	Log2FoldChange
*PFOS*	*TTN* *TBX18* *RSPO1*	1.29 2.42 2.24
*PFOA*	*TTN*	1.26
*PFNA*	*TTN* *MYH2* *ALDH1L2*	1.06 −2.03 4.21

## Discussion

In recent years we developed a 3D hiPSC-based assay, the PluriBeat, to assess the differentiation of EBs into beating cardiomyocytes (Lauschke et al. 2020). To overcome the labour-intensive visual detection of beating cells, we established the PluriLum, a standardized reporter gene assay using the same cell system, which measures activation of the cardiomyocyte-specific gene *NKX2.5* as a surrogate marker for cardiomyocyte differentiation (Lauschke et al. 2021; Treschow et al. 2024a). Previous studies showed that PFOS, PFOA, and GenX inhibit cardiomyocyte differentiation in both PluriBeat and the PluriLum assays (Treschow et al. 2024a). In the present study, we extend these findings by evaluating two additional PFAS, investigating their combined (mixture) effects, and exploring their mechanisms of action.

### Relative toxicity of the PFAS

As the PFAS class includes several thousand compounds, most of which lacking toxicity data (Patlewicz et al. 2026), there is growing interest in applying the concept of relative potency factors (RPFs) for their risk assessment, similar to the approach used for dioxin-like compounds. While recent studies have proposed RPFs for PFAS based on rodent data, it may be more appropriate to derive these factors using human-relevant models, such as the PluriLum assay, and compare them to previously derived values (Schrenk et al. 2020).

In the PluriLum assay, we observed varying embryotoxic potencies among the tested PFAS: PFNA and PFOS were more potent than PFOA and PFHxS. Using PFOA as the reference compound, the relative potencies based on IC_50_ values were 1: 5.2: 2.9: 0.4 for PFOA, PFNA, PFOS, and PFHxS, respectively. These values align reasonably well with previously reported RPFs from rodent studies. For instance, (Conley et al. 2022) found RPFs for PFOS relative to PFOA ranging from 1.8 to 43 depending on the developmental endpoint, with a value of 5.7 for reduced pup birth weight, very similar to our PFOS value of 5.2. (Bil et al. 2023) reported RPFs of 1: 5: 3: 0.6 and 1: 6: 2: 0.3 for the same PFAS based on liver and spleen weight in adult rats, respectively, which are remarkably consistent with our results for IC_50_ values, but slightly different from the RPFs based on BMC_10_ in our study (1: 2.3: 1.8: 0.8).

While direct comparisons between *in vitro* and *in vivo* systems must be interpreted cautiously, these parallel findings across diverse endpoints suggest that the PluriLum assay may be a promising tool for estimating the relative potencies of PFAS. Combined with physiologically based pharmacokinetic modelling, PluriLum could moreover support *in vitro* to *in vivo* extrapolation, enabling preliminary risk assessment of understudied PFAS and helping to prioritise them for further embryotoxic investigation.

### Mixture effects of PFAS

We observed that the effects of the balanced component-based mixtures (BMC_10_) of the four PFAS in the PluriLum assay aligned well with the predictions based on CA model. In contrast, the independent action (IA) model underestimated the observed responses, supporting the assumption of similar modes of action for these compounds. This finding is consistent with previous studies on PFAS mixtures. For example, *in vitro* studies on PPARα activation showed additive effects for a combination of PFOA, PFNA and PFOS, although some deviation from additivity was observed at high concentrations (> 64 µM), possibly due to synergism (Wolf et al. 2014). Another study using a PPARα activation assay found mixed outcomes, suggesting possible antagonism, but concluded that generalized CA provided the best fit to the observed effects (Nielsen et al. 2022). Similarly, a 3D liver spheroid study found that mixtures of 15 PFAS, including those tested here, followed CA below 25 µM, while synergistic effects emerged at higher concentrations (Addicks et al. 2023). In a neurodevelopmental assay based on neurite outgrowth, a mixture of four PFAS based on serum levels also followed CA, although the observed mixture effects were largely attributed to unspecific cytotoxicity of the individual PFAS (Ríos-Bonilla et al. 2024).

The response of the balanced mixture of the four PFAS closely followed the predicted CA model across the entire tested concentration ranges, with no deviations observed, unlike findings from previous studies (Wolf et al. 2014; Nielsen et al. 2022; Addicks et al. 2023). This suggests that under the conditions of the PluriLum assay, no interactions between the PFAS occurred. Overall, the evidence supports the use of CA as a default assumption for predicting PFAS mixture effects at low, environmentally relevant concentrations. These results indicate that the PluriLum assay may offer a reliable and practical tool for assessing mixture toxicity of the carboxylate and sulphonate subclass of PFAS.

The unbalanced mixtures, reflecting average PFAS exposure in European adults and children, did not induce a response in the PluriLum assay when comparing the nominal test concentrations to the actual human blood levels. Effects corresponding to nominal BMC_10_ levels were observed at high enrichment factors: approximately 685-fold in adults (total PFAS 23.3 µM) and 980-fold in children (total PFAS 17.6 µM), which might indicate a substantial margin of safety for embryotoxicity. However, we estimated that less than 2% of the added PFAS was taken up by the cells (discussed below) and moreover a substantial percentage of the PFAS are protein-bound, and therefore comparisons of nominal concentrations to actual human exposure levels does not make much sense.

### PFAS uptake in the PluriLum assay

PFAS quantification in exposed EBs revealed that PFOS had an uptake rate of 3.7- to 13.3-times higher than the other tested PFAS. Some PFAS are highly lipophilic and bind strongly to proteins, physicochemical properties that contribute to their high membrane permeability and bioaccumulation potential (Sanchez Garcia et al. 2018). The octanol/water partition coefficient (logK_OW_), a standard measure of lipophilicity, correlates with bioaccumulation, particularly for values above 5 (Mudlaff et al. 2024). While experimental logK_OW_ are limited for most PFAS, *in silico* models have predicted values exceeding 5 for all four studied PFAS, with PFOS ranking highest (logK_OW_ = 8.2), followed by PFNA (7.4), PFOA (6.9), and PFHxS (6.8) (Mudlaff et al. 2024), a ranking consistent with our observed uptake data.

Prior studies have shown that PFAS accumulation appears to be dependent on their affinity to phospholipids in lung epithelial cells and adipocytes (Sanchez Garcia et al. 2018), with PFOS accumulating more than PFOA in both cell types, while the shorter chain PFAS, perfluorobutane sulfonate and perfluorohexanoic acid, did not accumulate at all. The shorter chain of PFHxS and PFOA compared to PFOS and PFNA, respectively, may also partially explain why the latter ones appear to have a lower uptake rate than that of their longer chain homologues. Furthermore, (Shen et al. 2020) demonstrated that the four PFAS selected in our study can spontaneously penetrate a lipid bilayer model, with the sulfonates showing an increased ability to do so compared to the carboxylic acids. Importantly, it has been shown that PFOS and PFNA increase membrane fluidity to a greater extent than PFOA, while PFHxS appears to have no impact on this parameter (Hu et al. 2003; Xie et al. 2010; Fitzgerald et al. 2018; LaFond et al. 2023), which taken together, comes in line with the comparatively larger uptake of PFOS and PFNA in our cell model. Of note, a recent study conducted by Janssen et al. (Janssen et al. 2024) investigating the apical-to-basolateral membrane transport of PFAS, using three cell models of intestinal epithelium, showed that the ranking of uptake of these four PFAS followed the same ranking observed in our study. In this study, higher levels of PFOS were found in the cell lysates of all three models and low recovery on either basolateral or apical side, while PFHxS, closely followed by PFOA, showed the lowest recovery in the cellular portion of the membrane.

Reported protein binding for long-chain PFAS is typically > 97% and often > 99.9% depending on method, concentration, and species, and thus, the unbound free fraction is often very low (Ryu et al. 2024). Therefore, we attempted to estimate the percentage of PFAS that was actually retained in the EBs following repeated exposures, and we showed that the cellular fraction of PFAS was below 2% on Day 6. Nevertheless, these estimates should be interpreted with caution since our quantification method cannot distinguish between unbound PFAS present in the hollow core of the EBs or inside the cells, and membrane-bound PFAS that were released during precipitation with ACN, and thus, the fraction of free, active PFAS is expected to be even lower. Additionally, our calculations do not account for eventual losses of PFAS throughout the protocol due to, e.g., evaporation or plastic adhesion.

Importantly, when testing chemicals with different uptake rates, mixture effects might be affected, as these depend on internal levels and not on nominal concentrations. However, if there is no saturation of the cellular system, then the rate of uptake of each chemical in a mixture should be proportional to what is observed with individual chemical exposure. Under these conditions, if the chemicals act through the same mode of action and if no toxicokinetic interaction is expected, then concentration addition is a reasonable assumption (Escher et al. 2020). In fact, we assume these three conditions are fulfilled in our assay: no toxicokinetic interactions, same toxicological endpoint, and no saturation of the system, as shown by the low estimated fraction of cellular PFAS by Day 6. The fact that all three mixtures tested, either balanced or unbalanced, follow the concentration addition model for mixture effects is compatible with these assumptions.

### Transcriptomic effects of the PFAS

In this study, we evaluated the transcriptomic effects of PFAS exposure in EBs at BMC_10_ on Day 6 of cardiomyocyte differentiation in the PluriLum assay. RNA sequencing results were validated by RT-qPCR. Even when produced under identical conditions, EBs show high variability in cell-type proportions, spatial organization, and/or developmental timing. This may introduce biological noise and reduce statistical power. RNA sequencing of an EB measures the mean expression across all cells and thus, rare cell populations or subtle changes may become masked by dominant cell types. Consequently, strong transcriptomic differences may reflect compositional differences, not true gene-expression changes. Moreover, difficulty in interpreting gene-expression changes may arise, as it is not known whether e.g. an upregulation of a specific gene is due to an increased expression within a given cell type or due to a larger number of cells that express that gene. Thus, the results of our RNA sequencing analysis should be carefully interpreted.

Overall, we found relatively few DEGs for all four PFAS and PFHxS exposure did not produce any significant DEGs or enriched GO terms. However, PFHxS also showed the least uptake in EBs among the four PFAS and was the least potent on cardiomyocyte differentiation with a BMC_10_ of 31.4 µM. Thus, the intracellular concentrations of PFHxS might be too low to exceed the detection limits of the transcriptomic analysis. Moreover, PFHxS presented an outlier in one of the three replicates (shown in the PCA plot, Fig. 3b) which may also have affected the response. Alternatively, several other explanations could be involved such as PFHxS-induced effects caused by post-transcriptional or posttranslational regulation, post-translational modifications or altered protein activity, direct protein binding, metabolic interference, membrane effects, or adaptive homeostatic compensation – all factors that do not involve transcriptomic changes. The transcriptomic profiles of PFOA, PFNA and PFOS showed limited overlap, where the only gene commonly affected by PFOA, PFNA and PFOS was *TTN*, which encodes titin, a key protein for contractability in vertebrate striated muscles. These findings suggest a modest but specific impact of PFAS on cardiac development.

Our RNA sequencing analysis showed that PFOA, PFNA and PFOS enriched GO terms linked to cardiac development. PFOS in particular, deregulated multiple pathways related to cardiomyocyte and embryonic development, including canonical Wnt and BMP signaling. These findings are consistent with previous studies. For instance, PFOS has been shown to alter gene expression of *ISL1*, an early cardiac marker, in BiONi010-C cells at 25 µM (Davidsen et al. 2021). Moreover, using BiONi010-C and IMR90-1 hiPSC cell lines, Davidsen et al. observed reported enrichment in GO pathways related to cardiac development and function following PFOS exposure (Davidsen et al. 2022). Similar transcriptomic effects were also observed in human embryonic stem cells (hESC), with PFOS enriching GO terms related to cardiac differentiation (Yang et al. 2020; Qiu et al. 2024).

Our study showed that PFOA and PFNA enriched GO terms related to cardiac muscle development, including those associated with contractile fibers, sarcomeres, and their specific cellular components such as Z disc and I band. These findings suggest that PFOA may interfere with cardiac muscle development. Supporting this, Ko et al. reported that PFOA exposure reduced the expression of cardiac ion channel genes in hiPSC-derived cardiomyocytes (Ko et al. 2024)(Ko et al. 2024); while Davidsen et al. observed decreased gene expression of *MYH7*, a cardiac marker involved in muscle contraction, in BiONi010-C cells that were exposed to 100 µM PFOA (Davidsen et al. 2021). For PFNA, only zebrafish studies are available, which showed enrichment of KEGG pathways related to PPAR signaling and apoptosis (Gong et al. 2022; Qian et al. 2025). To our knowledge, no transcriptomic studies have explored the developmental effects or mechanisms of PFHxS.

Focal adhesion plays an important role in embryonic development, as the connection between cells and the extracellular matrix is essential for the formation and maintenance of organs (Wu 2007; Deng et al. 2025). Our results showed enriched GO terms related to focal adhesion after exposure to PFOA, PFNA, and PFOS, suggesting that exposure to these PFAS may affect organogenesis during embryonic development. The effects on GO terms for focal adhesion by PFAS are in line with previous studies in other cell lines e.g. the study by Chen et al. who showed that PFOS disrupted actin microfilaments and microtubule organization in Sertoli cells and that this effect could be rescued by overexpressing a focal adhesion kinase (Chen et al. 2017). Based on what is currently known about PFOS/PFOA cell biology, focal adhesion alterations in hiPSC-derived EBs may not be the primary molecular targets of PFAS, as PFAS-induced changes in focal adhesion gene expression are most likely secondary effects arising from either membrane perturbation, altered developmental signaling, metabolic/oxidative stress, nuclear receptor activation or differentiation shifts. Thus, the observed transcriptional changes in focal adhesion genes are probably downstream compensatory effects. Overall, our findings suggest that PFOA, PFNA and PFOS can impact embryonic and cardiomyocyte development and function through distinct molecular mechanisms.

## Conclusion

**Fig. 5:**
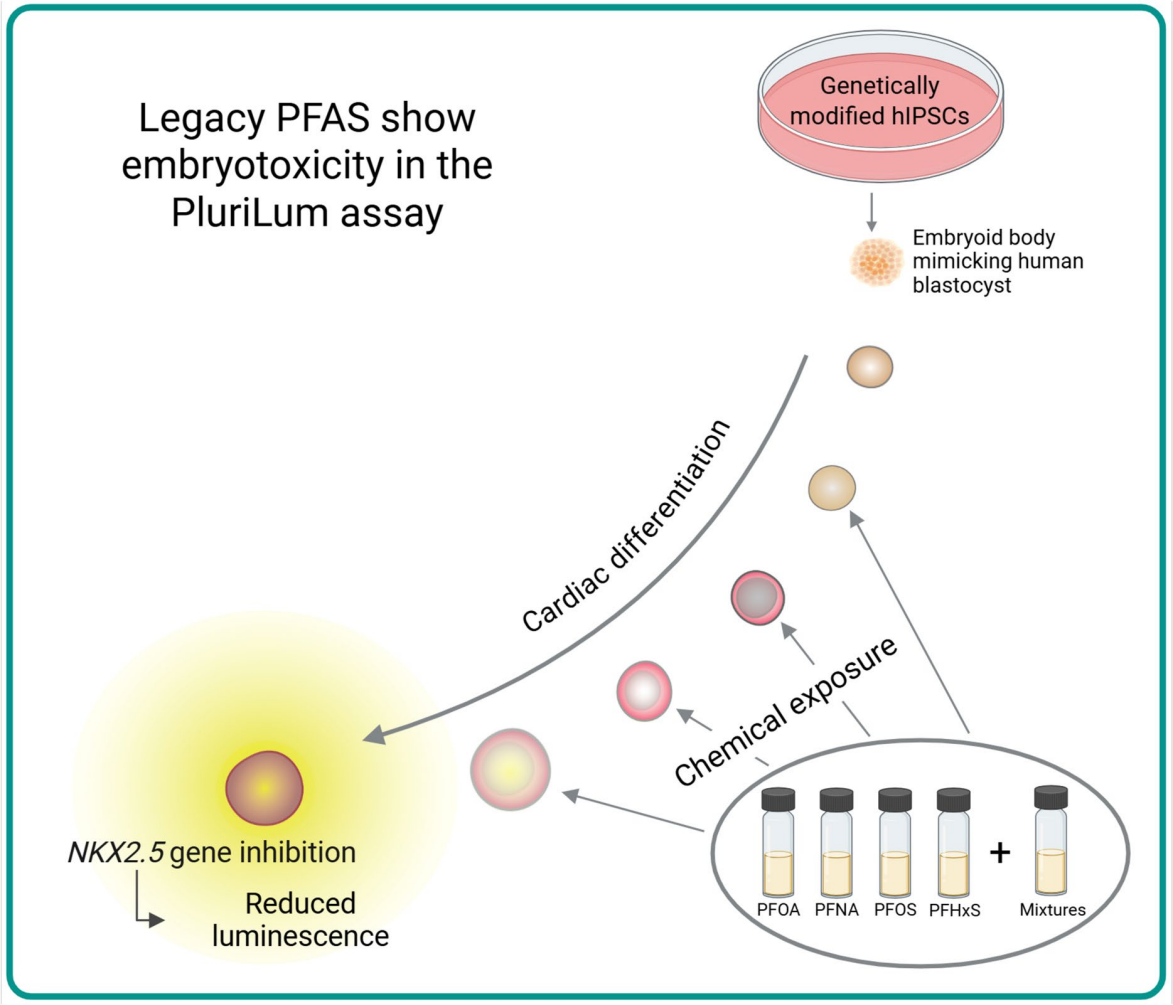
PFOA, PFNA, PFOS and PFHxS induced embryotoxicity in the PluriLum assay as measured by an inhibition of NKX2.5 activation

The embryotoxicity of four PFAS (PFOA, PFOS, PFNA and PFHxS), and their mixtures was evaluated using the PluriLum assay (Fig. 5). Individual compounds exhibited distinct potencies (PFNA > PFOS > PFOA > PFHxS), with relative potency factors aligning with those reported in other toxicity models. Mixtures of the four PFAS followed the principles of CA, and exposure scenarios based on average human serum levels elicited an effect in the assay. Less than 2% of the added PFAS could be found in the EBs and PFOS showed 3.7- to 13.3-fold higher cellular uptake compared to the other PFAS, likely due to its physicochemical properties and membrane interactions. Transcriptomic analysis revealed that PFNA, PFOS, and PFOA impacted pathways involved in embryonic, cardiomyocyte, and cardiac muscle development, underscoring the potential developmental toxicity of PFAS.

## Supplementary information

Below is the link to the electronic supplementary material.

ESM 1 (XLSX 15.9 KB)

ESM 2 (XLSX 47.5 KB)

ESM 3 (XLSX 89.8 KB)

## Data Availability

Data is available from the corresponding author upon reasonable request. RNA sequencing data is available in: https://zenodo.org/records/17600724.
